# Intrathecal immune checkpoint inhibitors as adjunctive therapy for leptomeningeal disease: A case series and systematic review

**DOI:** 10.1007/s10143-026-04454-z

**Published:** 2026-08-21

**Authors:** Manav Daftari, Christian K. Ramsoomair, Anurag Aka, Vratko Himic, Anna Hudson, Manuela Aramburu Berckemeyer, Morgan Johnson, Victor M. Lu, Jay Chandar, Vaidya Govindrajan, Jose Lutzky, Michael E. Ivan, Ricardo J. Komotar, Ruham Nasany, Ashish H. Shah

**Affiliations:** 1 Department of Neurological Surgery, University of Miami Miller School of Medicine, Miami, FL USA; 2 Sylvester Comprehensive Cancer Center, University of Miami Miller School of Medicine, Miami, FL USA; 3 Section of Virology and Immunotherapy, Department of Neurosurgery, Leonard M. Miller School of Medicine, University of Miami, Miami, FL 33136 USA

**Keywords:** Leptomeningeal Disease, Immune Checkpoint Inhibitors, Intrathecal Therapy, Systematic Review, Safety, Efficacy

## Abstract

Immune checkpoint inhibitors (ICIs) are promising for leptomeningeal disease (LMD), but systemic delivery is limited by poor cerebrospinal fluid penetration and treatment-related toxicity. This study evaluates the safety and efficacy of adjunctive intrathecal (IT) ICI therapy compared with systemic ICI alone. We report two institutional cases of melanoma-associated LMD treated with IT ICI alongside systemic therapy. Following PRISMA guidelines, PubMed, Embase, and Scopus were systematically reviewed, identifying 28 eligible studies consisting of case reports, small series, and early phase trials. Of 542 patients screened, 201 received ICI therapy: 161 systemic ICI alone and 40 IT ICI with concurrent systemic therapy. Patient-level and aggregate data were extracted to compare adverse events (AEs), progression-free survival (PFS), and overall survival (OS). Exploratory time- and dose-adjusted analyses accounted for differences in treatment exposure. IT ICI therapy demonstrated a favorable safety profile. At the patient level, adjunct IT delivery was associated with a non-significant trend toward reduced grade ≥ 3 AE risk (RR 0.50; 95% CI, 0.22–1.13; *p* = 0.096). At the event level, adjunct IT therapy was significantly associated with 72% fewer grade ≥ 3 AEs per patient (rate ratio 0.28; 95% CI, 0.12–0.64; *p* = 0.003). Dose-adjusted meta-regression suggested cumulative exposure did not explain these differences. No significant differences were observed in PFS or OS after multivariable adjustment. Adjunct IT ICI therapy was not associated with increased adverse event risk or high-grade toxicity burden compared with systemic ICI alone, supporting its feasibility as an adjunctive therapeutic strategy for LMD that warrants prospective evaluation.

**Supplementary Information:**

The online version contains supplementary material available at 10.1007/s10143-026-04454-z.

## Introduction

Leptomeningeal disease (LMD) is defined as dissemination of tumor cells into the leptomeninges and cerebrospinal fluid (CSF). LMD is a fatal complication that occurs in up to 15% of patients with primary solid tumors, most commonly melanoma, lung cancer, and breast cancer [1–4]. Despite treatment, median survival remains three to six months, highlighting the urgent need for more effective therapeutic strategies [5]. 

Immune checkpoint inhibitors (ICIs) have transformed solid tumor management by inducing robust tumor microenvironmental changes [6–12]. However, systemically delivered ICIs face substantial barriers in treating LMD. The restrictive blood-brain barrier (BBB) limits monoclonal antibody diffusion into the CSF. Although systemic ICIs can exert intracranial effects through peripheral T-cell activation, responses are often blunted by treatment resistance, T-cell exhaustion, and the immunosuppressive tumor microenvironment [13–18]. 

Intrathecal (IT) delivery of ICIs represents a promising strategy to enhance LMD treatment by achieving direct BBB penetration. This approach achieves higher local concentrations at lower doses, owing to the small CSF volume, and limited drug metabolism, potentially prolonging antibody half-life [19]. Importantly, most patients with LMD receive multimodal systemic therapy including targeted agents, chemotherapy, or systemic ICIs [20]. As such, IT ICI therapy represents a promising adjunct to systemic targeted therapy in patients with LMD.

In this combined case series and systematic review, we primarily evaluate the safety of IT ICI therapy as an adjunct to systemic therapy compared with systemic ICI alone in patients with LMD. We first present two institutional cases of metastatic melanoma–associated LMD treated with adjunct IT ICIs to review our institutional experience. We then conduct a systematic review of published studies to evaluate safety as the primary outcomes and efficacy as a secondary outcome.

## Methods

### Institutional case series

With IRB approval, retrospective chart review was performed for two patients receiving IT ICI therapy at our institution. Demographics, diagnosis, IT ICI dosing, concurrent therapies, adverse events (AEs), and survival outcomes were collected.

### Systematic review

A systematic search of PubMed, Embase, and Scopus was performed following PRISMA guidelines. No date limits were applied. Reference lists and clinical trial registries were also screened. Search terms included: “immune checkpoint inhibitors,” “immune checkpoint blockade,” “anti-PD-1,” “anti-PD-L1,” “anti-CTLA-4,” “pembrolizumab,” “nivolumab,” “atezolizumab,” “durvalumab,” “ipilimumab”, “leptomeningeal disease,” “leptomeningeal metastasis”, “intravenous,” “systemic,” “intracranial,” “intrathecal,” “Ommaya reservoir,” “CSF delivery,” and/or “intracerebroventricular”. Inclusion criteria required: (1) LMD confirmed by CSF cytology or MRI; (2) administration of at least one ICI; (3) reporting of AEs and/or survival outcomes. Exclusion criteria: preclinical studies, reviews, conference abstracts without full text, duplicate datasets, or studies lacking patient counts. Individual patient-level data (IPD) was extracted when available; study-level aggregate data was included for AE analysis. Studies were screened independently by two reviewers with disagreements resolved by a third.

Cohort Definitions and Outcomes. Patients were assigned to the IT-adjunct cohort if they received IT ICI with concurrent systemic therapy (IV ICI, targeted therapy, or chemotherapy); the systemic cohort included patients receiving systemic ICI without IT administration. The primary outcome was AEs graded per CTCAE; secondary outcomes included overall survival (OS) and progression-free survival (PFS).

Risk-of-bias and methodological-quality assessments were performed using design-specific tools: JBI checklists for case reports and case series, MINORS for prospective single-arm studies, and ROBINS-I V2 for retrospective nonrandomized intervention-effect cohorts [21–24]. 

*Adverse Events Analysis*. AEs were classified by administration route. For the IT-adjunct cohort, AEs attributed to the combined adjunct IT and concurrent systemic treatment regimen were recorded as reported by each study; for the systemic cohort, AEs attributed to systemic ICI therapy were recorded as reported by each study. When available, the number of patients experiencing AEs and total events were recorded, along with grade 1–2 and grade ≥ 3 events per CTCAE criteria. AE frequency was standardized by dividing total events by patients at risk. Study-specific incidence rates were log-transformed and pooled using random-effects meta-analysis. Pooled rate ratios comparing adjunct IT versus systemic administration were obtained with 95% confidence intervals using Poisson-based variance estimates. Heterogeneity was quantified using τ² and I². For patient-level analysis, proportions experiencing ≥ 1 AE or ≥ 1 grade ≥ 3 AE were pooled using random-effects models; relative risks were estimated using log-transformed effects. To assess potential confounding by ICI drug class, sensitivity AE analyses were performed using only systemic comparator studies with PD-1 inhibitor-only regimens. Exploratory analyses accounted for treatment-exposure differences between cohorts. Time-adjusted incidence rates were calculated as events per patient-month, and dose-adjusted meta-regression evaluated whether cumulative ICI exposure explained observed toxicity differences.

*Efficacy Analysis*: PFS (time from ICI initiation to progression) and OS (time from LMD diagnosis to death or last follow-up) were assessed using IPD when available. Patients alive at last follow-up were censored. Multivariable Cox proportional hazards regression was performed adjusting for tumor histology and time from diagnosis to treatment initiation.

All analyses were performed in R (v4.3.3) using metafor and dplyr packages; *p* < 0.05 was considered significant.

##  Results

### Case series

**Fig. 1:**
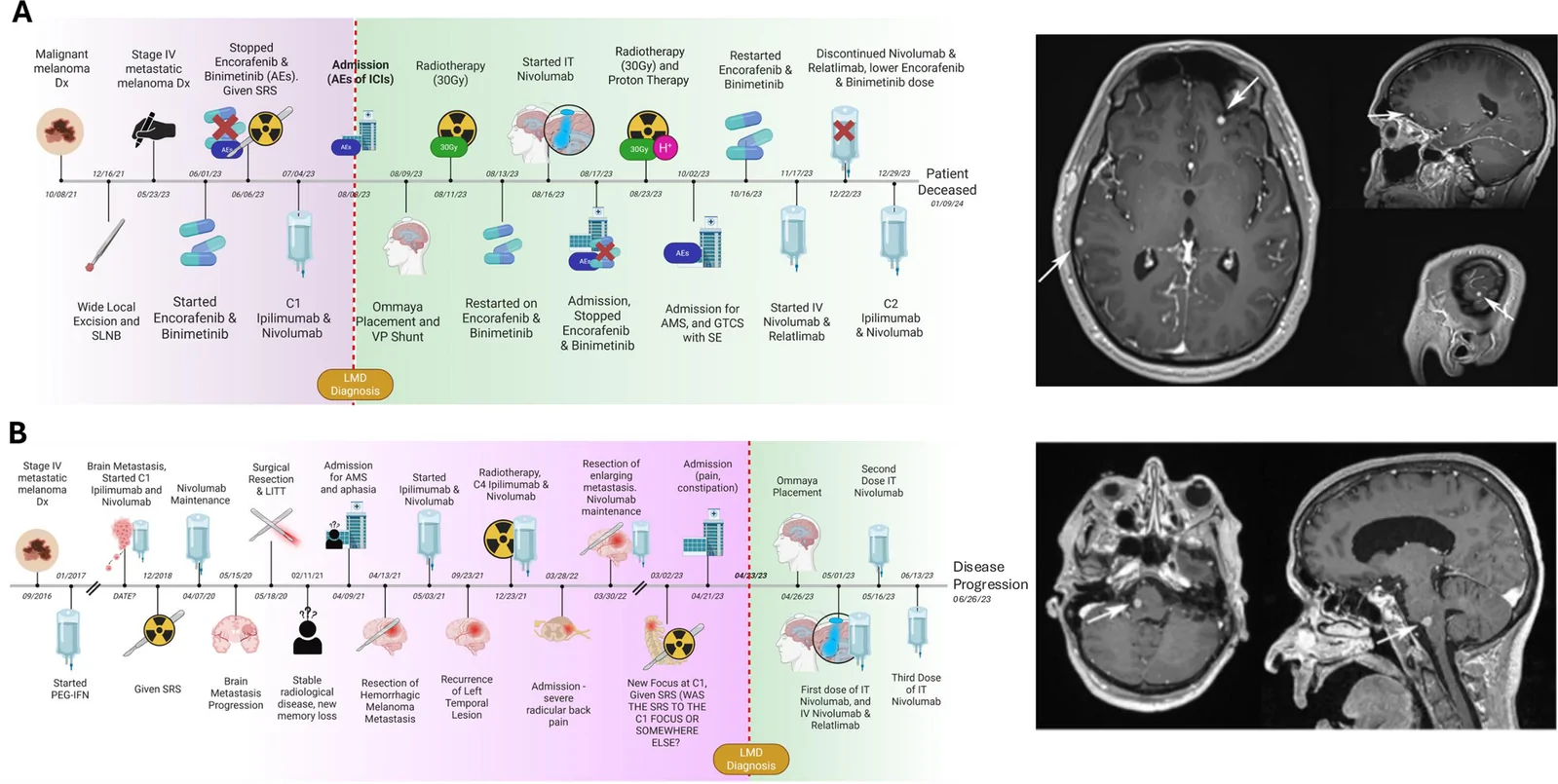
Detailed treatment timeline and representative magnetic resonance imaging (MRI) of Patient 1 (**A**) and Patient 2 (**B**)

*Case #1.* A 38-year-old man with BRAF-mutant metastatic melanoma, initially treated with surgical excision, developed stage IV disease with brain metastases two years after diagnosis. He received targeted therapy (encorafenib/binimetinib) and Gamma Knife radiosurgery, then transitioned to IV ipilimumab/nivolumab. Given elevated intracranial pressure, a ventriculoperitoneal (VP) shunt and Ommaya reservoir were placed. Five days post-operatively, he received IT nivolumab (50 mg). The VP shunt was closed prior to IT therapy and remained closed until 5–7 days post-treatment to optimize CSF drug retention. Within 24 h, he developed nausea, altered mental status, and disorientation consistent with grade 1–2 immune-mediated encephalitis, which resolved with corticosteroid therapy. Given this AE, IT ICI was discontinued as a precautionary measure. He was subsequently maintained on systemic therapy, including targeted agents and later IV nivolumab/relatlimab, until disease progression. He died from disease progression 5 months after diagnosis. PFS from IT ICI was approximately 1 month; OS from LMD diagnosis was approximately 5 months. A complete treatment timeline is presented in Fig. 1A.

*Case #2.* A 38-year-old woman with metastatic melanoma of the left thigh, initially treated with surgical excision and pegylated interferon achieving complete regression, later developed recurrent intracranial disease. Between 2018 and 2022, she received multiple therapies including systemic ipilimumab/nivolumab, stereotactic radiosurgery, surgical resections, and laser interstitial thermal therapy (LITT), followed by maintenance systematic nivolumab. In April 2023, she presented with worsening back pain and was found to have new leptomeningeal enhancement on MRI; CSF cytology confirmed LMD. An Ommaya reservoir was placed, and she received IT nivolumab (50 mg) while continuing systemic immunotherapy. IT ICI was well tolerated without AEs, and during combined IT and systemic ICI therapy, the patient experienced clinical improvement including reduced back pain and improved ambulation. She received three IT doses and was maintained on combined IT and systemic ICI until disease progression 8 weeks later. PFS from IT ICI was approximately 2 months. She was subsequently lost to follow-up, precluding the assessment of OS. A treatment timeline is presented in Fig. 1B.

Given the promising yet heterogeneous results, we sought to evaluate these findings at a larger scale that would be more amenable to systematic analysis. Accordingly, we conducted a systematic review of LMD patients treated with IT ICIs across three published literature databases.

### Systematic review characteristics

A total of 94 studies were screened, with 28 included (Supplementary Fig. 1). Across these studies, 542 patients were screened, and 201 ICI-treated patients were analyzed (Supplementary Fig. 2): 161 received systemic ICI alone and 40 received IT ICI with concurrent systemic therapy. Age and sex were similar between cohorts (Table 1), but primary malignancies differed: the systemic cohort included skin (45.3%), lung (22.9%), and breast cancer (19.3%), whereas the IT-adjunct cohort was predominantly melanoma (97.5%) with one breast cancer patient (2.5%). ICI regimens also varied; systemic patients received PD-1 inhibitors, CTLA-4 inhibitors, or nivolumab–ipilimumab, while all IT patients received PD-1 inhibitors (Table 2).

**Table 1:** Baseline demographic, tumor, and clinical characteristics of patients with leptomeningeal disease stratified by treatment route

Characteristics (no. patients for whom information is available)	Systemic	IT-Adjunct	*p*-value
Cohort Size (no.)	161	40	
Demographics			
Median Age (years)	46.5 (37.5–61.5) (*n* = 12)	49 (40–55) (*n* = 7)	0.66
Sex, female, (%)	40% (*n* = 20)	47.5% (*n* = 40)	
Primary Tumor			
Skin Cancer (melanoma & SCC)	109	39	
Breast Cancer	32	1	
Lung Cancer (all subtypes)	41	-	
Gastrointestinal Tract Malignancy	7	-	
Central Nervous System Malignancy	7	-	
Renal Clear Cell Carcinoma	1	-	
Bladder Cancer	1	-	
Ovarian Cancer	2	-	
Head and Neck SCC	1	-	
Primary Hepatic NET	1	-	
Cancer of Unknown Primary	1	-	
Median Time from 1º Diagnosis to LMD (months)	24 (6.25–49.2) (*n* = 10)	72 (24–84) (*n* = 7)	0.35
LMD Diagnosis Methods			
T1 Contrast MRI (%)	77.5 (*n* = 80)	100 (*n* = 40)	
CSF cytology (%)	*n* = 80	*n* = 40	
Positive	70 (*n* = 56)	57.5 (*n* = 23)	
Negative	11.3 (*n* = 9)	40 (*n* = 16)	
Not Tested	18.8 (*n* = 15)	2.5 (*n* = 1)	
Duration of ICI treatment (months)	6.0 (2.1–11.5) (*n* = 12)	2.0 (1.0–4.0) (*n* = 7)	0.054
Time from LMD Diagnosis to ICI treatment	1.45 (0–4.3) (*n* = 12)	0 (0–2.0) (*n* = 7)	0.16

*Systemic* systemic immune checkpoint inhibitor therapy, *IT* intrathecal immune checkpoint inhibitor therapy, *LMD* leptomeningeal disease, *SCC* squamous cell carcinoma, *NET* neuroendocrine tumor, *CNS* central nervous system, *CSF* cerebrospinal fluid, *MRI* magnetic resonance imaging. Continuous variables are presented as median (interquartile range).

**Table 2:** Distribution of ICI drug classes and concomitant therapies by treatment route

ICI Drug Class (*n*)	Systemic	IT-Adjunct
PD-1 inhibitor	69	40
CTLA-4 inhibitor	12	-
PD-L1 inhibitor	4	-
LAG-3 inhibitor	1	-
CTLA-4 + PD-1 (Comb.)	21	-
Unspecified	54	-
Concomitant Drug Type (n)		
IV Immune Checkpoint Inhibitor	–	39
Radiotherapy	18	–
Cytotoxic Chemotherapy	8	1
Targeted Therapy	6	4

Immune checkpoint inhibitors (ICIs) include programmed cell death protein-1 (PD-1) inhibitors (e.g., nivolumab, pembrolizumab, and unspecified PD-1 inhibitors) and cytotoxic T-lymphocyte–associated protein-4 (CTLA-4) inhibitors. Targeted therapy includes BRAF inhibitors (BRAFi), combined BRAF/MEK inhibitors (B/M), anti–vascular endothelial growth factor (VEGF) therapy (e.g., bevacizumab), and other specified or unspecified targeted agents. Cytotoxic chemotherapy includes agents with direct cytotoxic activity, such as methotrexate (MTX), temozolomide (TMZ), pemetrexed, and intrathecal topotecan. Concomitant therapies were defined as treatments administered concurrently with ICI therapy.

Methodological appraisal results are summarized in Supplementary Fig. 3. Case reports/series were generally adequately reported by JBI criteria, while single-arm studies had expected limitations related to lack of concurrent controls. Retrospective intervention-effect cohorts had serious ROBINS-I V2 concerns, primarily due to confounding by indication, treatment-selection bias, and overlapping therapies.

### Safety analysis

**Fig. 2:**
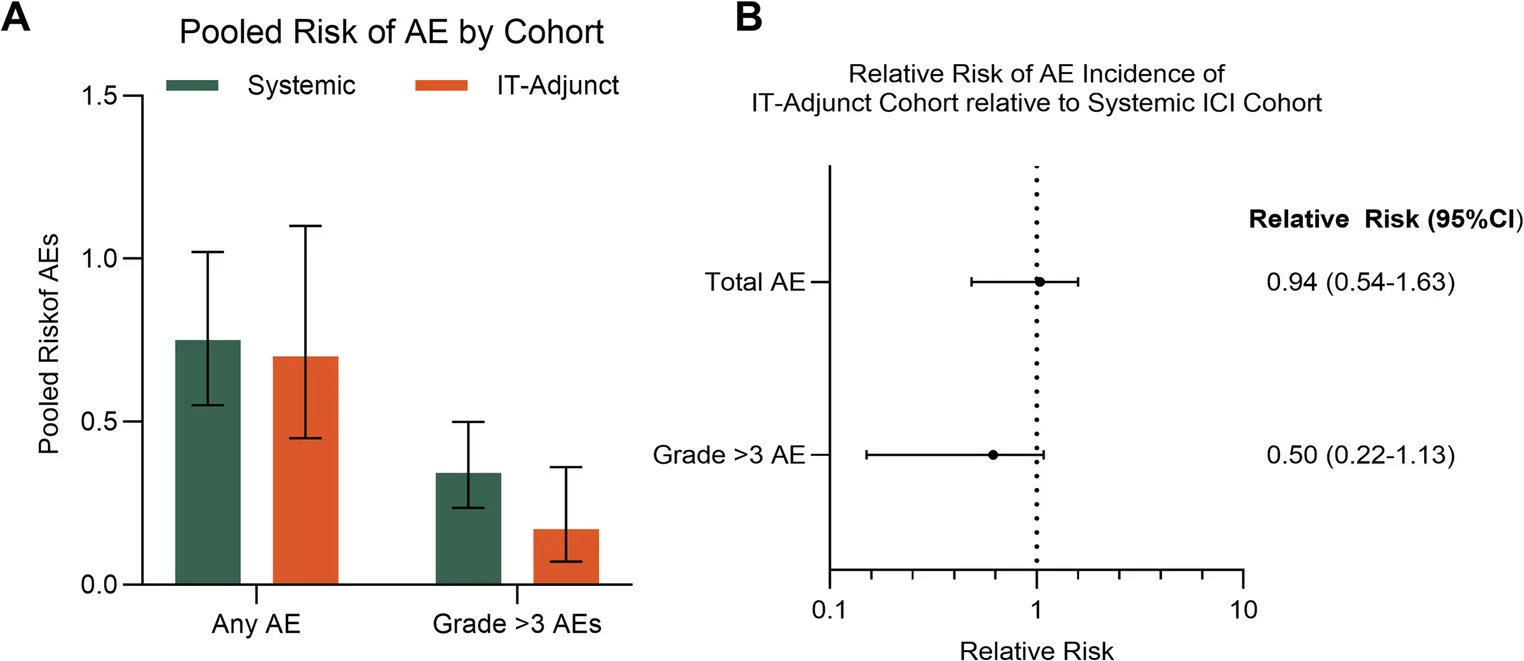
Patient-level adverse event risk comparing systemic and intrathecal-adjunct ICI therapy cohorts

*Patient-level analysis.* The pooled risk of an LMD patient experiencing any AE was 0.70 (95% CI, 0.45–1.10) in the IT-adjunct cohort compared with 0.75 (95% CI, 0.55–1.02) in the systemic cohort **(**Fig. 2A**)**. The relative risk was 0.94 (0.54–1.63; *p* = 0.83). For grade ≥ 3 AEs, the pooled risk was 0.17 (0.08–0.36) amongst the IT-adjunct cohort and 0.34 (0.24–0.50) for the systemic cohort. The relative risk was 0.50 (0.22–1.13; *p* = 0.096) (Fig. 2B). Between-study heterogeneity was moderate for any AE (I²=36–63%) but negligible for grade ≥ 3 events (I²=0%). To account for any AE differences associated with ICI type heterogeneity, we also performed a sensitivity analysis restricted to PD-1 inhibitor-only systemic comparator studies. Patient-level grade ≥ 3 AE risk remained numerically lower with IT-adjunct therapy but was not statistically significant (RR 0.52, 95% CI 0.20–1.33).

**Fig. 3:**
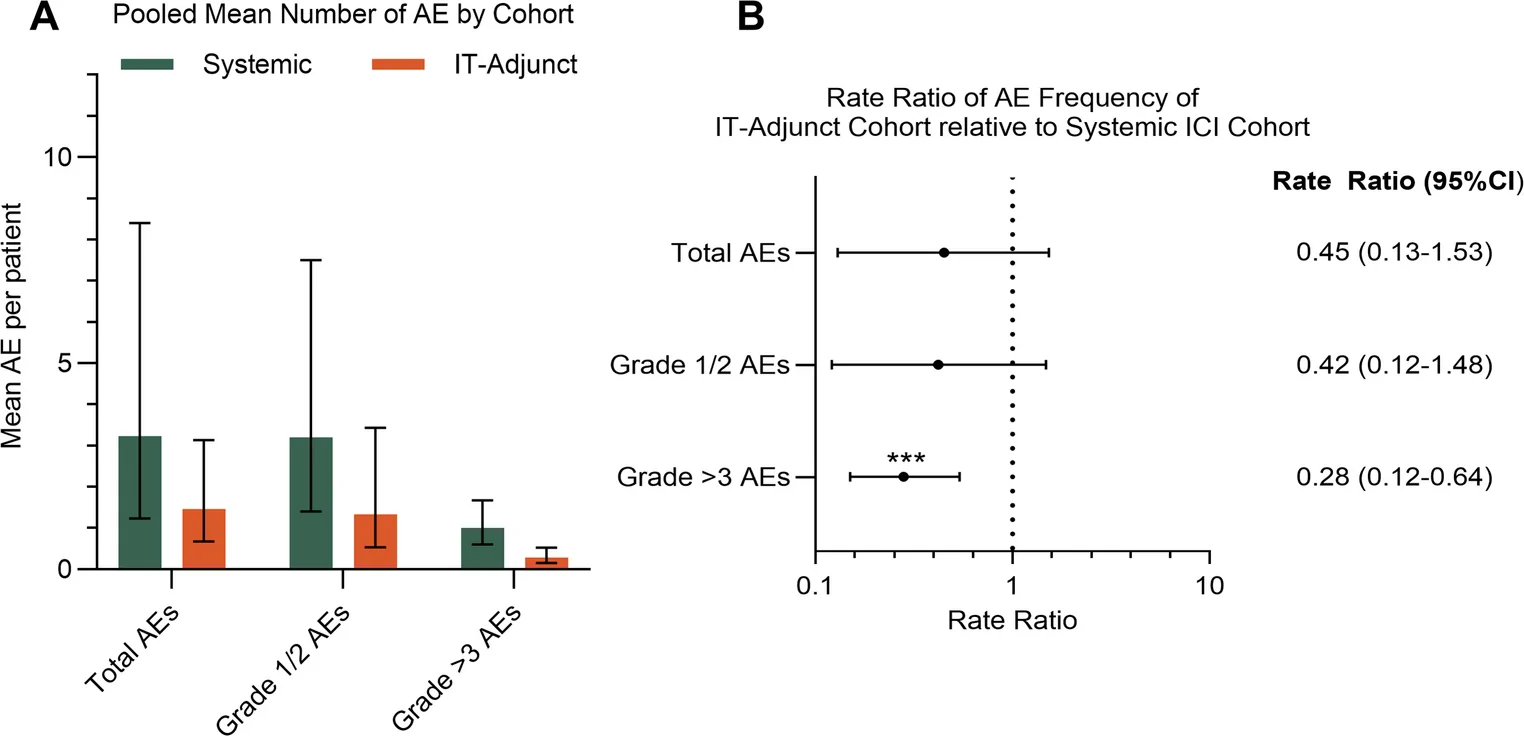
Event-level adverse event frequency and rate ratios comparing systemic and intrathecal-adjunct ICI therapy cohorts

*Event-level analysis*. To complement patient-level AE risk estimates, we performed an event-level analysis to compare toxicity burden between cohorts. The pooled incidence of total AEs was 1.46 events per patient (0.68–3.13) in the IT adjunct cohort versus 3.23 events per patient (1.24–8.40) in the systemic cohort **(**Fig. 3A**).** For grade 1–2 AEs, incidence was 1.33 events per patient (0.52–3.42) for IT versus 3.20 (1.37–7.50) for systemic therapy. For grade ≥ 3 AEs, IT therapy demonstrated 0.28 events per patient (0.15–0.54) compared with 1.00 events per patient (0.60–1.67) for systemic therapy. The rate ratio was 0.28 (0.12–0.64; *p* = 0.003), indicating a significant 72% reduction in high-grade toxicity incidence with adjunct IT administration **(**Fig. 3B**).** Between-study heterogeneity ranged from moderate to substantial (I² = 53–98%). Again, accounting for AE heterogeneity due to ICI class, we found this result persisted when the systemic comparator was restricted to PD-1 inhibitor-only studies, with a lower grade 3/4 AE event rate in the IT-adjunct cohort (rate ratio 0.26, 95% CI 0.10–0.68; Supplementary Fig. 4).

To account for differences in treatment duration, exploratory time-adjusted analyses were performed using patient-months of exposure. After normalization, AE rates were similar between IT-adjunct and systemic cohorts for total AEs (0.177 vs. 0.201 events/patient-month; RR 0.88, *p* = 0.922), grade 1–2 AEs (0.086 vs. 0.149; RR 0.58, *p* = 0.749), and grade ≥ 3 AEs (0.013 vs. 0.057; RR 0.22, *p* = 0.362). These findings suggest that shorter treatment duration in the IT-adjunct cohort partly contributed to lower unadjusted AE rates. Because shorter duration also reduces cumulative drug exposure, dose-adjusted meta-regression was performed to assess whether cumulative ICI dose explained these safety differences. No significant dose association was observed for any AEs (*p* = 0.873, I²=59.5%) or grade ≥ 3 AEs (*p* = 0.294, I²=0.0%), suggesting that the observed difference in route-of IT delivery is not entirely attributable to cumulative dose alone and may reflect route-specific differences including treatment duration (Supplementary Figs. 5–6).

### Efficacy analysis

Median PFS was 6.0 months in the systemic cohort and 7.0 months in the IT adjunct cohort (log-rank *p* = 0.11; HR 0.42; 95% CI, 0.09–1.87). Median OS was 9.0 months in the systemic cohort and 5.0 months in the IT-adjunct cohort (log-rank *p* = 0.38; HR 0.60; 95% CI, 0.14–2.65). After multivariable adjustment for histology and time to ICI initiation, no statistically significant differences in PFS or OS were observed between cohorts. However, the small IT cohort and missing patient survival cannot guarantee non-inferiority in efficacy (Supplementary Figs. 7–8).

## Discussion

ICI therapy has transformed cancer therapy, yet its safety and efficacy in LMD remain incompletely understood. At our institution, we report two cases of patients treated with adjuvant IT ICI therapy. One patient developed mild corticosteroid-responsive AEs after a single dose, while the other well-tolerated multiple doses with transient neurological improvement and no AEs. These two cases illustrate the heterogeneity of clinical outcomes with IT ICI, including both early toxicity-related discontinuation and tolerability with transient clinical benefit, and are presented as descriptive institutional experience. Given this heterogeneity, we sought to contextualize these observations within the broader published literature by conducting a systematic review.

At the patient level, IT-adjunct therapy showed a favorable trend toward reduced grade ≥ 3 AE risk (RR 0.50, *p* = 0.096). Event-level analysis further supported this signal, demonstrating a significant (rate ratio = 0.28, *p* = 0.003) reduction in grade ≥ 3 AE incidence. This finding persisted in a sensitivity analysis restricted to systemic PD-1 inhibitor-only comparator studies (rate ratio = 0.26, 95% CI 0.10–0.68), suggesting that the observed event-rate difference was not solely driven by differences in ICI class. Time-adjusted analyses showed that AE differences were attenuated after normalizing for treatment duration, suggesting that the shorter ICI treatment duration of IT-adjunct cohort partly contributed to the lower AE rates. Interestingly, dose-adjusted meta-regression indicated that cumulative ICI exposure alone did not explain the observed safety differences. This supports a potential route-specific advantage of adjunct IT delivery through localized CSF distribution and reduced systemic exposure. Notably, these exploratory analyses were limited by incomplete reporting and use of surrogate exposure estimates.

Overall, adjunct IT administration was not associated with increased adverse event risk or high-grade toxicity burden compared with systemic therapy alone. Exploratory efficacy outcomes were comparable, with median PFS of 7.0 months for IT adjunct versus 6.0 months for systemic therapy, and median OS of 5.0 versus 9.0 months, respectively, without significant differences. These results should be cautiously interpreted within the context of treatment heterogeneity, as IT therapy consisted exclusively of PD-1 inhibitors and was often used in salvage settings, whereas systemic therapy included broader ICI regimens across more varied clinical contexts. Additionally, these findings remain vulnerable to residual confounding from tumor-histology imbalance and limited patient-level data. Taken together, these findings support the feasibility of IT ICI as an adjunctive strategy that does not appear to add meaningful toxicity to existing systemic regimens.

### Current clinical use of IT delivery

Intrathecal delivery is well-established in clinical practice for chemotherapeutics, antimicrobials, and analgesics [25–27]. For oncologic indications, IT chemotherapy is routinely used in LMD arising from both primary brain tumors and systemic malignancies to bypass the BBB and deliver higher drug concentrations to the CSF [14, 28, 29]. Methotrexate (MTX), cytosine arabinoside, and corticosteroids are common agents. In fact, IT MTX achieves up to 100-fold higher CSF concentrations compared to systemic dosing [16]. In patients with LMD from HER2-negative metastatic breast cancer, improved overall survival has been hypothesized to result from enhanced CSF drug distribution and improved tolerability [29]. Optimized regimens employing smaller, more frequent IT doses have further been found to improve therapeutic response while limiting toxicity [14, 30, 31]. 

While IT administration can be achieved via either lumbar puncture or intraventricular Ommaya reservoir, most clinical protocols for repeated ICI or chemotherapy delivery favor Ommaya systems [32–34]. Ommaya reservoirs enable consistent, reproducible dosing and superior drug distribution throughout the CSF, though they require neurosurgical implantation and carry risks such as infection or catheter malfunction [29, 34, 35]. 

### Safety

Despite ICI therapy showing promise in treatment of primary cancers including lung cancer, breast cancer, melanoma and others [6–10], these survival benefits come with notable risks. ICIs are associated with a spectrum of immune-related adverse events (irAEs), which can range from mild to life-threatening [36, 37]. The pathogenesis of these irAEs is multifactorial and not yet fully understood [38]. 

In our institutional cohort, the occurrence of immune-mediated encephalitis following a single dose of IT nivolumab warrants careful consideration. Neurological irAEs, though rare with systemic ICI (< 1% grade ≥ 3), carry among the highest fatality rates of irAEs [39]. Encephalitis can present very early, with a reported median onset of 3.0 treatment cycles, and may manifest as meningoencephalitis or focal encephalitis with distinct prognostic implications [40, 41]. Direct introduction of ICI into the CSF compartment, while bypassing the BBB and potentially advantageous for antitumor efficacy, may lower the threshold for CNS-directed autoimmune toxicity, a concern supported by the 20–40% incidence of chemical aseptic meningitis with conventional IT chemotherapy [42]. Notably, however, the encephalitis in Case 1 developed within 24 h of IT nivolumab administered just five days after VP shunt and Ommaya reservoir placement, in a patient with ongoing systemic immune activation from IV ipilimumab/nivolumab. The temporal proximity to surgery and pre-existing systemic immune priming suggest that post-operative neuroinflammation and prior systemic ICI exposure may have contributed to the observed toxicity, rather than IT delivery alone. Although the encephalitis resolved with corticosteroids, consistent with NCCN management recommendations, the potential for severe CNS injury cannot be excluded [43]. These observations highlight that both the timing of IT ICI relative to neurosurgical procedures and the degree of concurrent systemic immune activation may play a role in CNS neurotoxicity risk, and underscore the need for dedicated neurological monitoring, low thresholds for neuroimaging and CSF analysis, and standardized neurotoxicity grading with predefined stopping rules in prospective trials.

To further contextualize the safety of IT ICI therapy, we examined preclinical and clinical studies, which demonstrated IT-adjunct ICI delivery appears to have a favorable safety profile compared with systemic administration. In murine models, IT anti-PD1 caused no morbidity, severe neurologic symptoms, or histologic evidence of neurologic injury within 14 days [44]. Clinically, the first-in-human phase I trial of IT nivolumab in melanoma LMD reported no grade 3/4 events with IT treatment alone, whereas sequential IV ICI was associated with occasional grade 3/4 toxicities, including lymphopenia, elevated liver enzymes, headache, and fatigue [32]. Consistent with our findings, this IT/IV cohort had a lower overall grade 3–4 toxicity rate than prior IV-only ICI studies in LMD, and a retrospective melanoma LMD series similarly reported only mild events, such as headache and back pain [32]. However, existing IT ICI data remains limited to PD-1 inhibitors, underscoring the need to evaluate broader ICI targets and combination strategies.

The relative safety of IT administration likely reflects the pharmacokinetic properties of monoclonal antibodies. With molecular weights exceeding 140,000 Daltons, ICIs exhibit limited penetration across the blood-CSF barrier, achieving CSF concentrations of only approximately 1% of serum levels following systemic administration [45]. Consequently, IV dosing requires high systemic drug exposure to achieve therapeutic CNS concentrations, increasing the risk of peripheral immune activation and associated adverse events [46, 47]. In contrast, IT administration delivers ICIs directly into the CSF compartment, achieving therapeutic local concentrations while minimizing systemic exposure. This compartmentalization may limit the activation of autoreactive T cells outside the CNS, thereby reducing the incidence of systemic immune-related adverse events.

### Efficacy

Preliminary data suggests that IT ICI delivery demonstrates potential for treating LMD by providing concentrated ICI doses, limiting systemic toxicities, and potentially overcoming resistance mechanisms seen with systemic therapy. In patients with melanoma LMD, IT nivolumab achieved a median OS of five months, even among heavily pretreated individuals, and produced durable responses in select cases [32]. However, many patients in early studies received only limited treatment, often a single dose, emphasizing the need to optimize dosing intensity and frequency while also exploring new therapeutic classes and combination approaches. While systemic ICI delivery achieves only minimal penetration into the CSF (CSF-serum ratios of 0.33%−1.9% for nivolumab and less than 1% for pembrolizumab have been reported), they can still induce PD-1 depletion in CSF-infiltrating T cells [45, 48, 49]. These findings confirm target engagement despite low BBB penetrance and low absolute concentrations in the CSF [45]. IT administration, by contrast, provided higher local antibody concentrations in the CSF, potentially leading to stronger T-cell activation, enhanced effector function, and improved antitumor immunity within the CNS compartment.

### Limitations

This study is limited by small sample sizes, heterogeneous study designs, and reliance on case reports and early-phase trials. Formal methodological appraisal similarly supports interpreting the evidence as hypothesis-generating. Variability in concurrent therapies, dosing schedules, and outcome reporting complicates cross-study comparisons. Concurrent radiotherapy was reported in a subset of systemic ICI patients but not in the IT-adjunct cohort, introducing potential confounding in toxicity comparisons. Because IT ICI is administered alongside systemic therapy in clinical practice, this comparison evaluates adjunctive IT ICI rather than monotherapy, precluding isolation of IT-specific effects.

Second, AE analyses relied on aggregate study-level reporting, with the systemic cohort serving as an indirect comparator rather than a within-study control. Event-rate analyses could not distinguish repeated AEs within the same patient and are best interpreted as aggregate toxicity burden. Incomplete reporting of baseline demographics, including age and treatment duration, further limited matched comparisons between cohorts. Substantial between-study heterogeneity was observed in event-level analyses (I² = 53–98%), reflecting differences in tumor histology, ICI regimen, concomitant therapies, AE reporting practices, and follow-up duration. Random-effects meta-analysis was used to account for this variance, and multiple sensitivity analyses were performed to evaluate robustness; however, event-level findings should be interpreted as hypothesis-generating.

Third, cohort composition differed substantially: the IT-adjunct cohort received only anti-PD-1 therapy and consisted almost exclusively of melanoma patients, whereas the systemic cohort included mixed histologies and broader ICI regimens. Route-attributed differences therefore cannot be fully separated from tumor-specific treatment patterns and ICI class effects. Although PD-1 inhibitor-only sensitivity analyses and multivariable survival adjustment were performed, residual confounding cannot be excluded.

Fourth, the shorter median OS in the IT-adjunct cohort (5.0 vs. 9.0 months) may reflect selection bias, as this cohort had a substantially longer interval from primary diagnosis to LMD (72 vs. 24 months), consistent with a more heavily pretreated salvage population. Baseline performance status was inconsistently reported and could not be compared. Survival comparisons should be considered hypothesis-generating.

Finally, CSF cytology was negative in 40% of IT-adjunct patients versus 11.3% in the systemic cohort, raising the possibility that some IT-adjunct patients were diagnosed on MRI findings alone and may have had less established disease at baseline, potentially influencing both AE rates and efficacy outcomes. Prospective trials with standardized diagnostic criteria and protocols are needed to clarify these findings.

## Conclusion

LMD remains a rapidly progressive and often fatal complication of advanced solid tumors, with therapeutic options have been limited by poor CNS penetration and systemic toxicities. In this combined case series and systematic review, adjunct IT ICI therapy was not associated with increased adverse event risk or high-grade toxicity burden compared with systemic ICI alone. These findings suggest that the addition of IT ICI to systemic therapy does not appear to confer excess toxicity, supporting its potential as a feasible adjunctive therapeutic strategy for LMD. Larger prospective studies are needed to define optimal dosing, combinations, patient selection, and long-term outcomes, but emerging evidence supports IT ICI therapy as a promising strategy for LMD.

## Supplementary Information

Below is the link to the electronic supplementary material.

Supplementary File 1 (PDF 614 KB)

Supplementary File 2 (PDF 448 KB)

Supplementary File 3 (PDF 431 KB)

## Data Availability

No datasets were generated or analysed during the current study.

## Declarations

### Clinical trial number

This study was a retrospective analysis; therefore, a clinical trial registration number is not applicable.
