# Supplementary material for: Intrathecal immune checkpoint inhibitors as adjunctive therapy for leptomeningeal disease: A case series and systematic review
Source: Neurosurg Rev. 2026 Aug 21;49(1):531. doi: 10.1007/s10143-026-04454-z (PMC13493459; doi:10.1007/s10143-026-04454-z)
Supplement: Supplementary file 2 [file 10143_2026_4454_MOESM2_ESM.pdf]

Supplementary Figures

Title: Intrathecal Immune Checkpoint Inhibitors as Adjunctive Therapy for Leptomeningeal Disease: A Case Series and Systematic Review

Authors: Manav Daftari[1], Christian K Ramsoomair[1], Anurag Aka[1], Vratko Himic[1], Anna Hudson[1], Manuela Aramburu Berckemeyer[1], Morgan Johnson, [1] Victor M. Lu[1], Jay Chandar[1], Vaidya Govindrajan[1], Jose Lutzky[2], Michael E. Ivan[1], Ricardo J. Komotar[1], Ruham Nasany[2], Ashish H. Shah[1, 2]

[1] Department of Neurological Surgery, University of Miami Miller School of Medicine, Miami, FL, USA

[2] Sylvester Comprehensive Cancer Center at the University of Miami Miller School of Medicine, Miami, FL, USA

Corresponding Author

Name: Ashish H. Shah

Section of Virology and Immunotherapy,

Department of Neurosurgery,

Leonard M. Miller School of Medicine,

University of Miami, Miami, FL 33136, USA.

Email: ashah@med.miami.edu

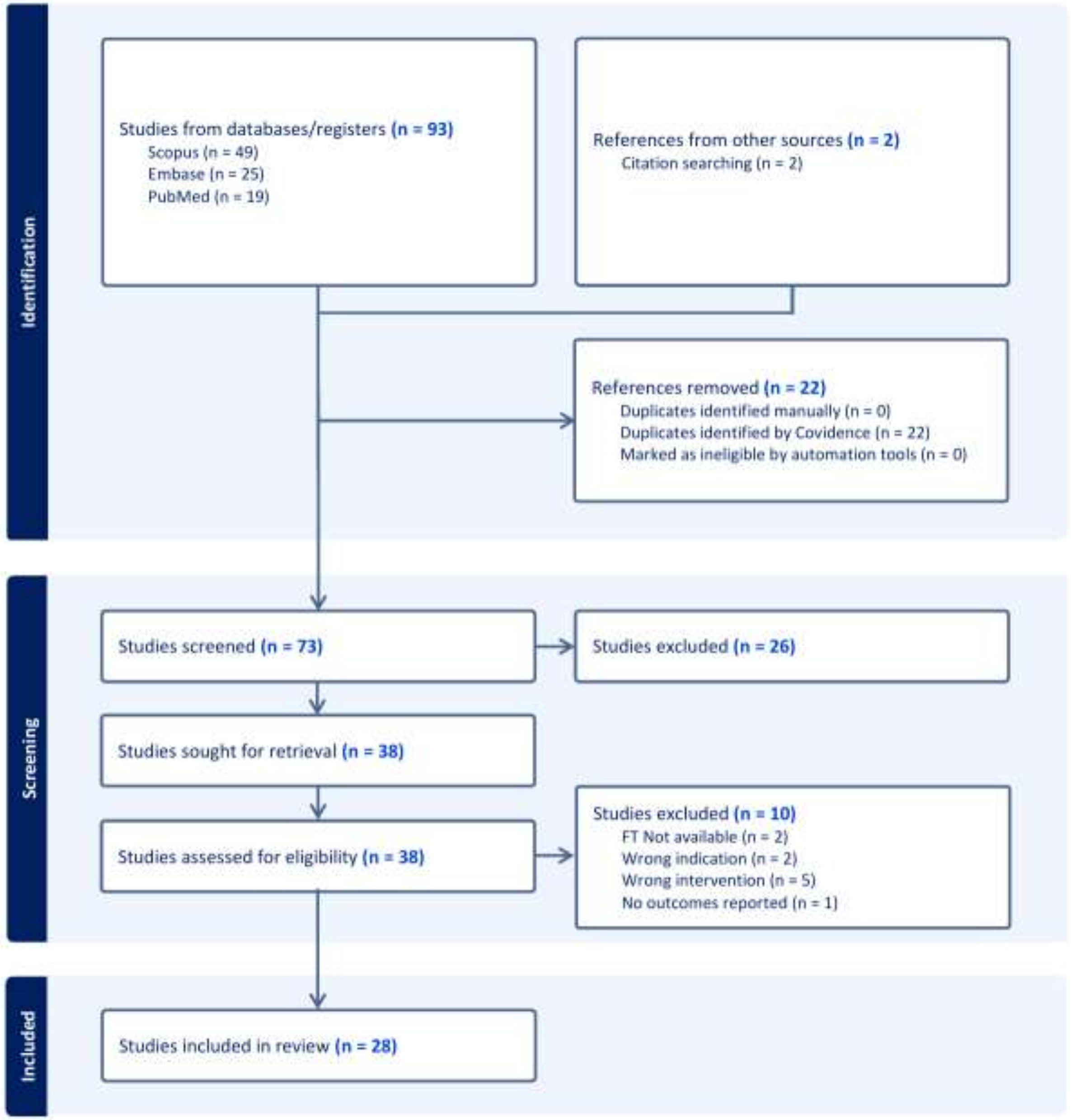

Supplemental Figure 1. PRISMA Flow Diagram

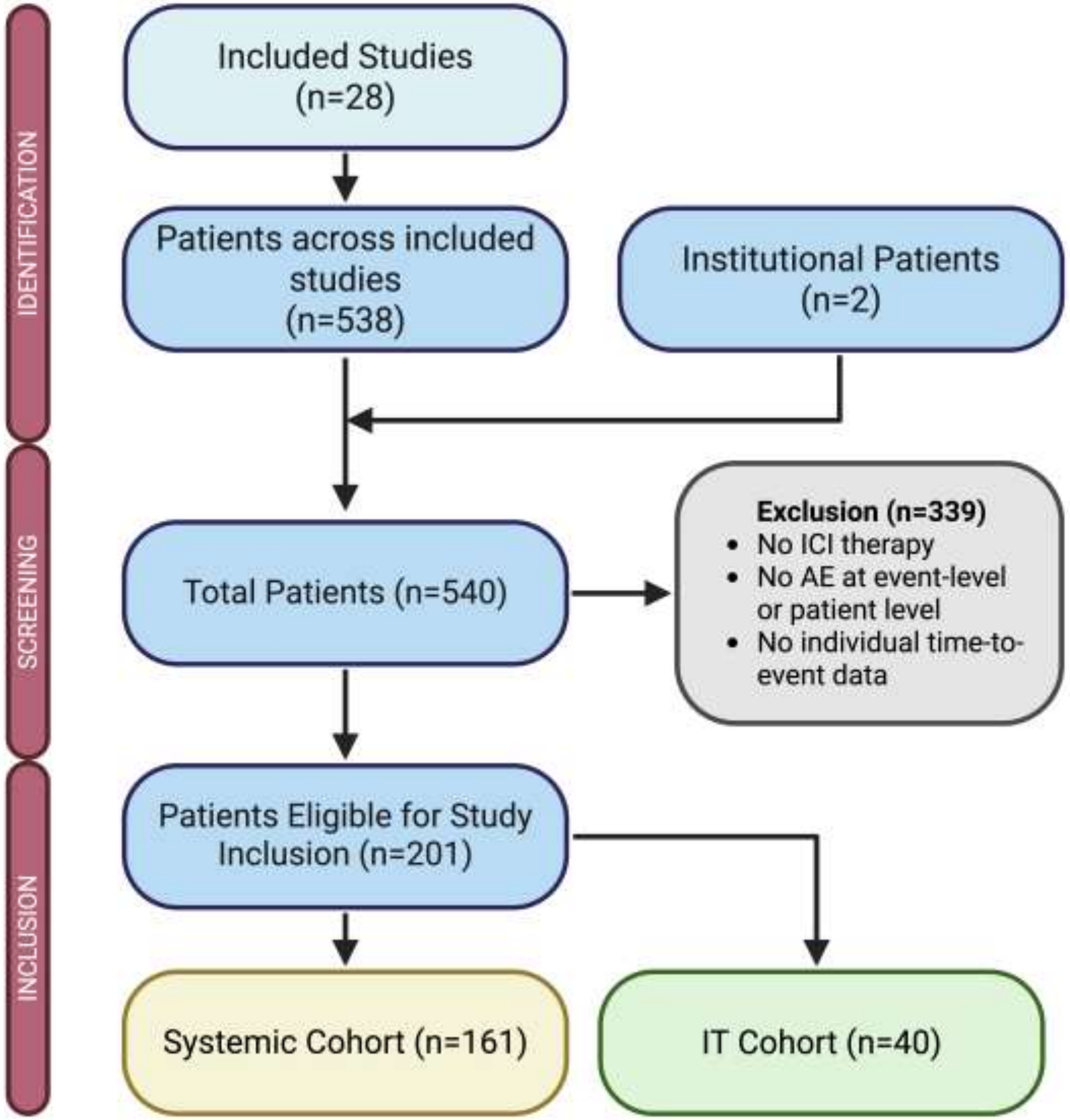

Supplemental Figure 2. Flow diagram of patient identification, screening, and cohort assignment

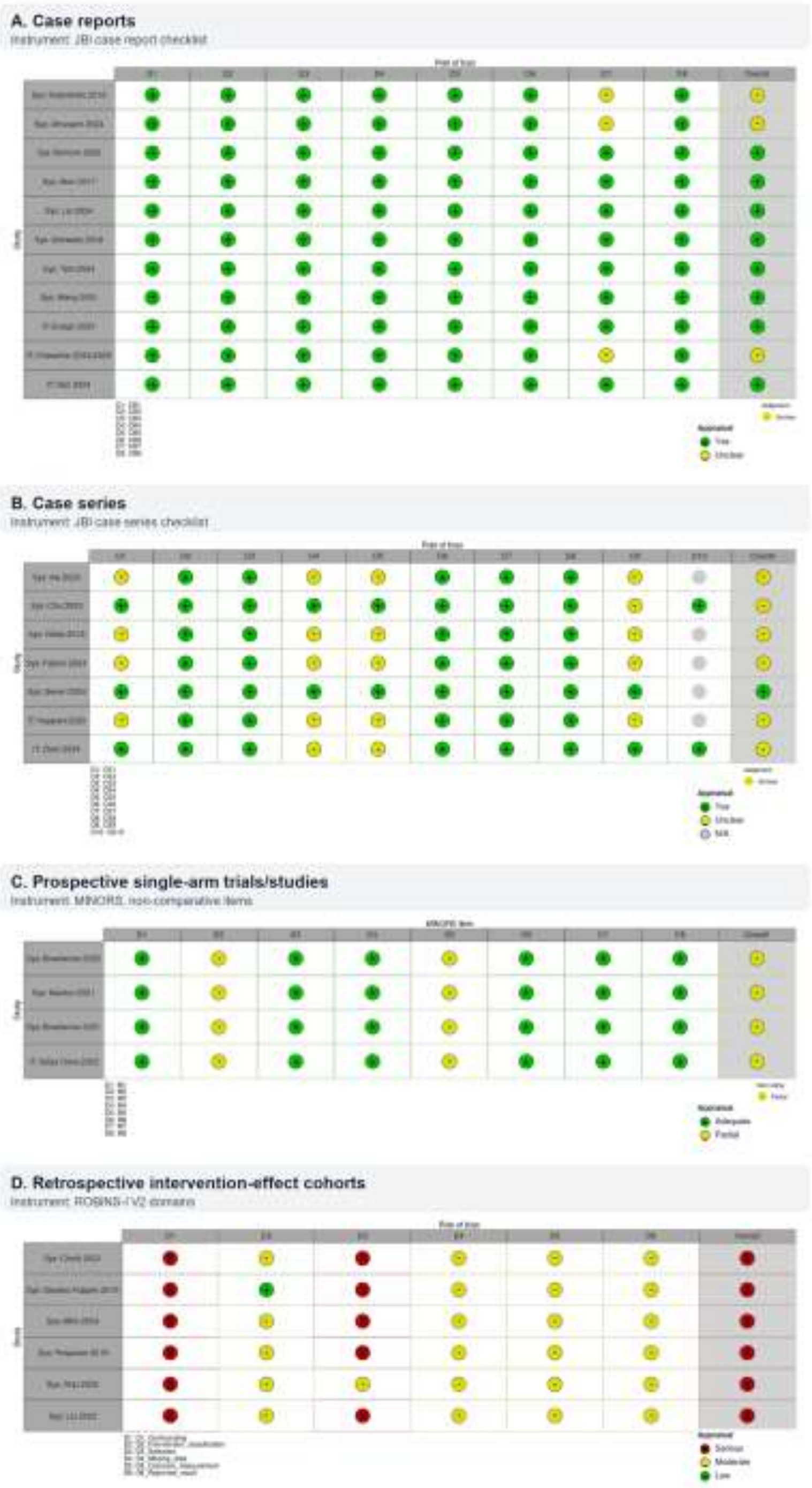

Supplemental Figure 3. Methodological appraisal and risk-of-bias assessment by study type

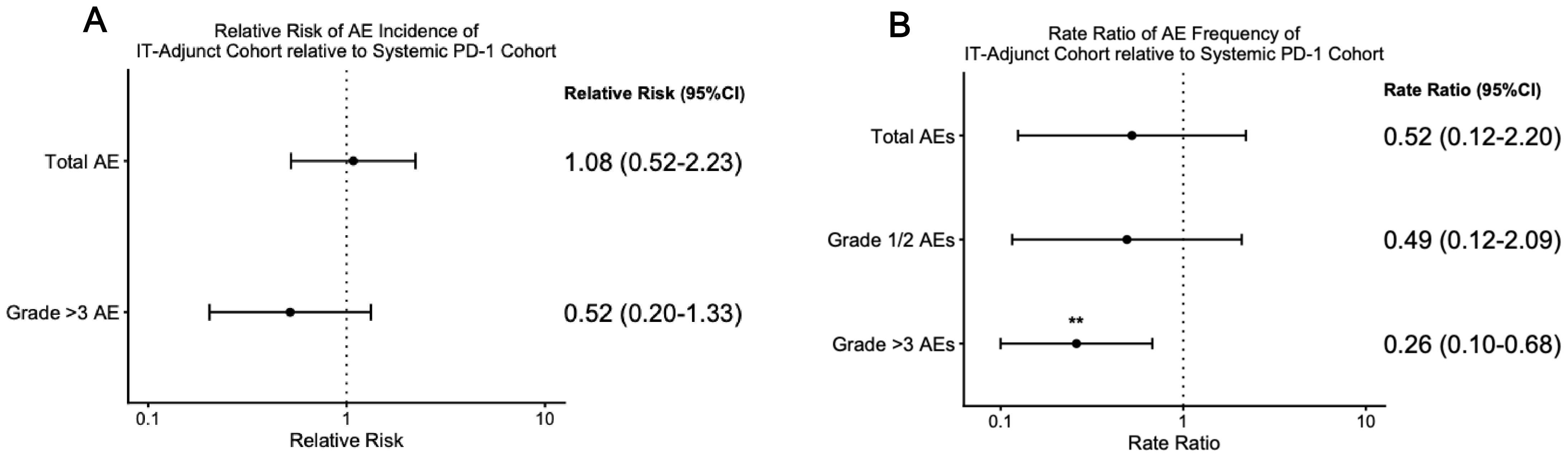

Supplemental Figure 4. PD-1 inhibitor-only sensitivity analysis of AE outcomes at (A) patient level and (B) event level

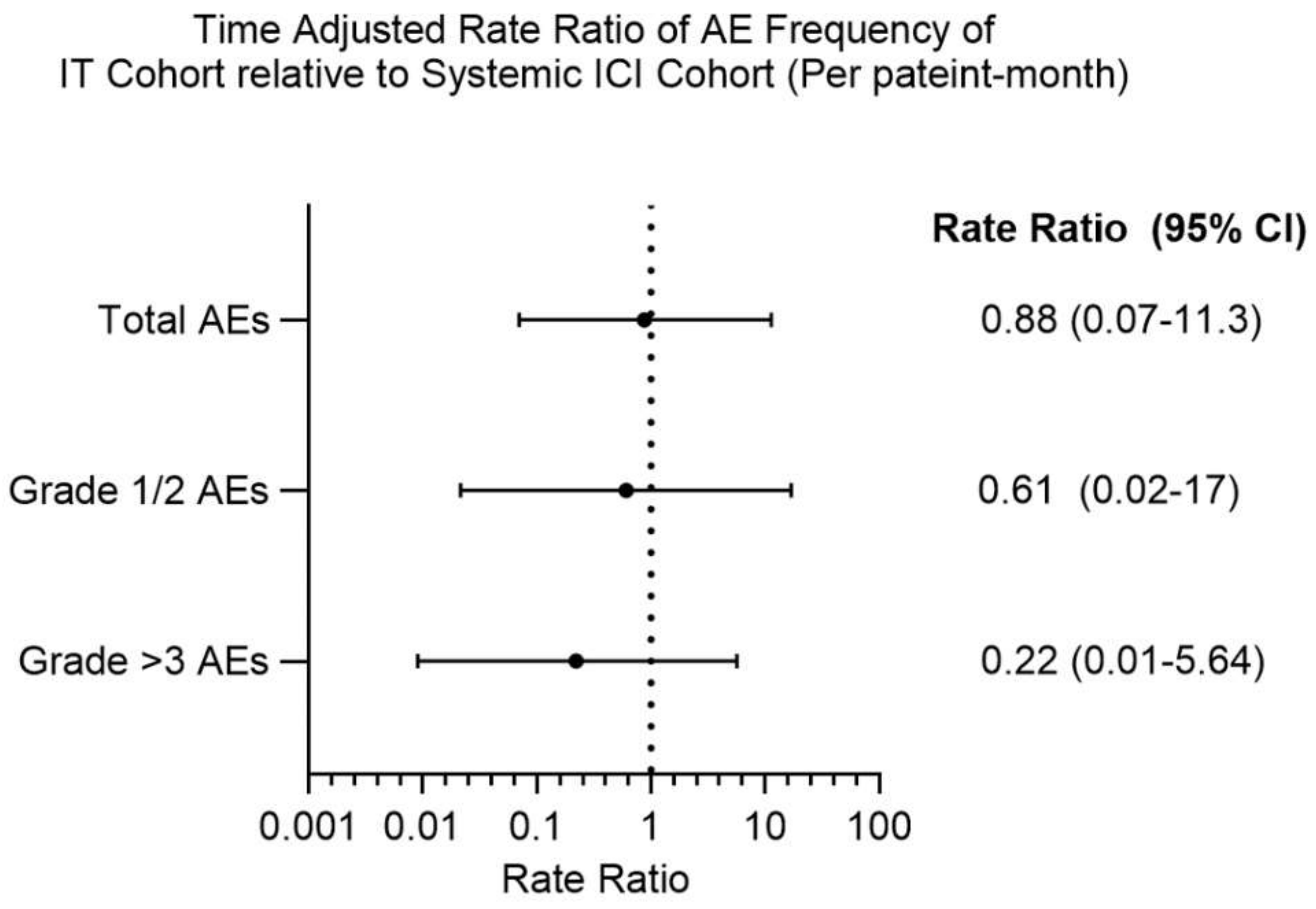

Supplemental Figure 5. Time-adjusted rate ratios of AEs comparing IT and systemic cohorts

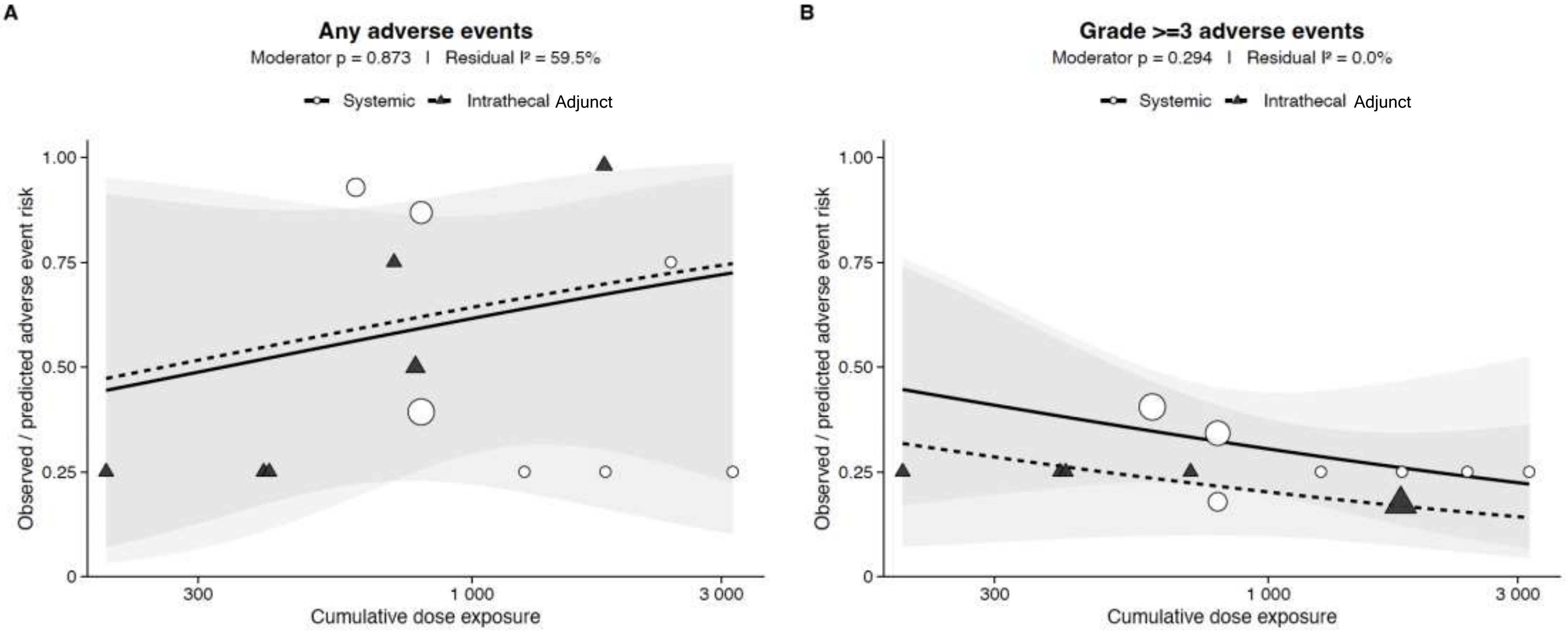

Supplemental Figure 6 . Dose-adjusted meta-regression of AE risk by treatment route

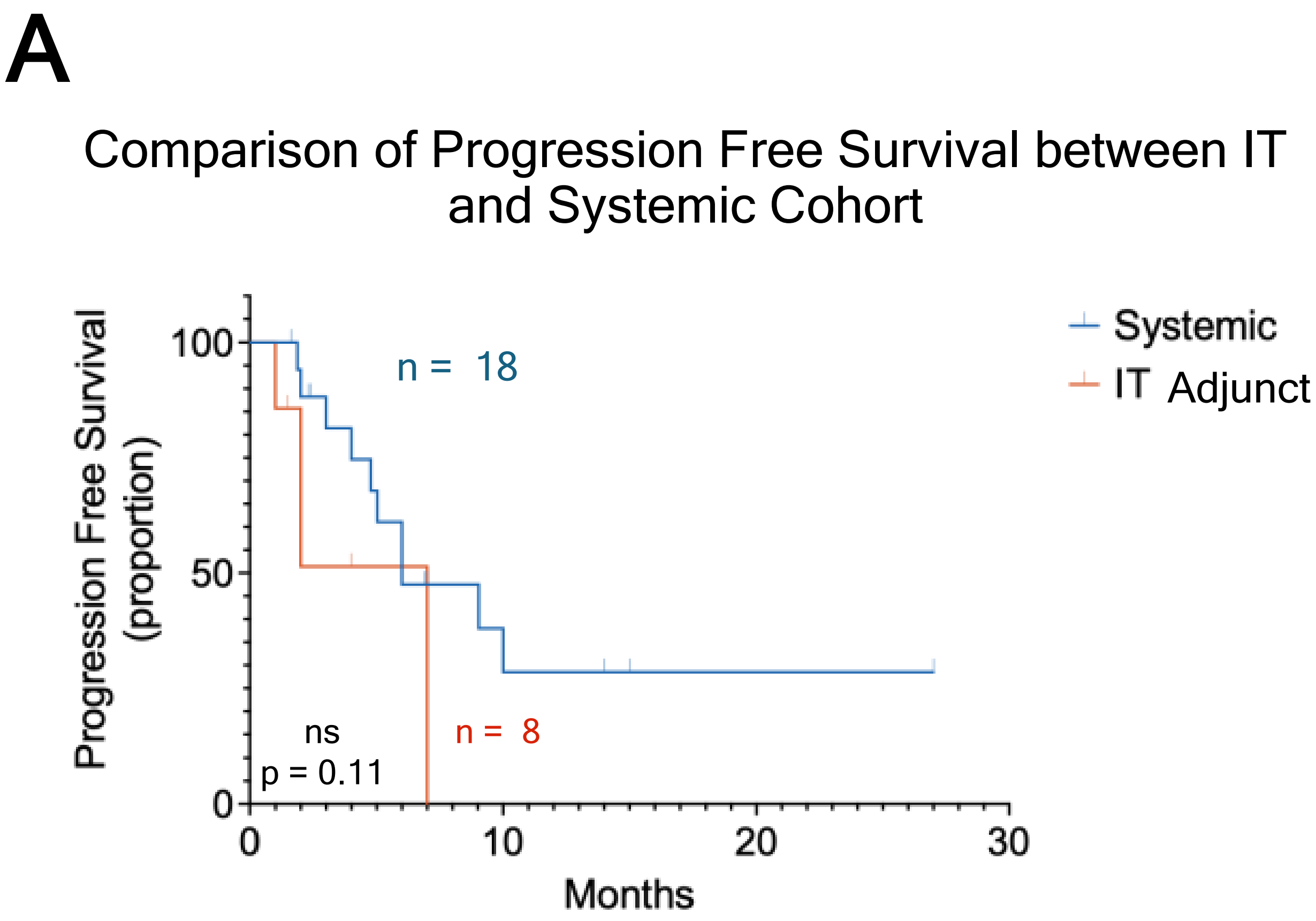

Number at Risk

Time (mos)	0	3	6	9	12	24
Systemic	18	12	9	5	3	1
IT Adjunct	7	2	1	0	0	0

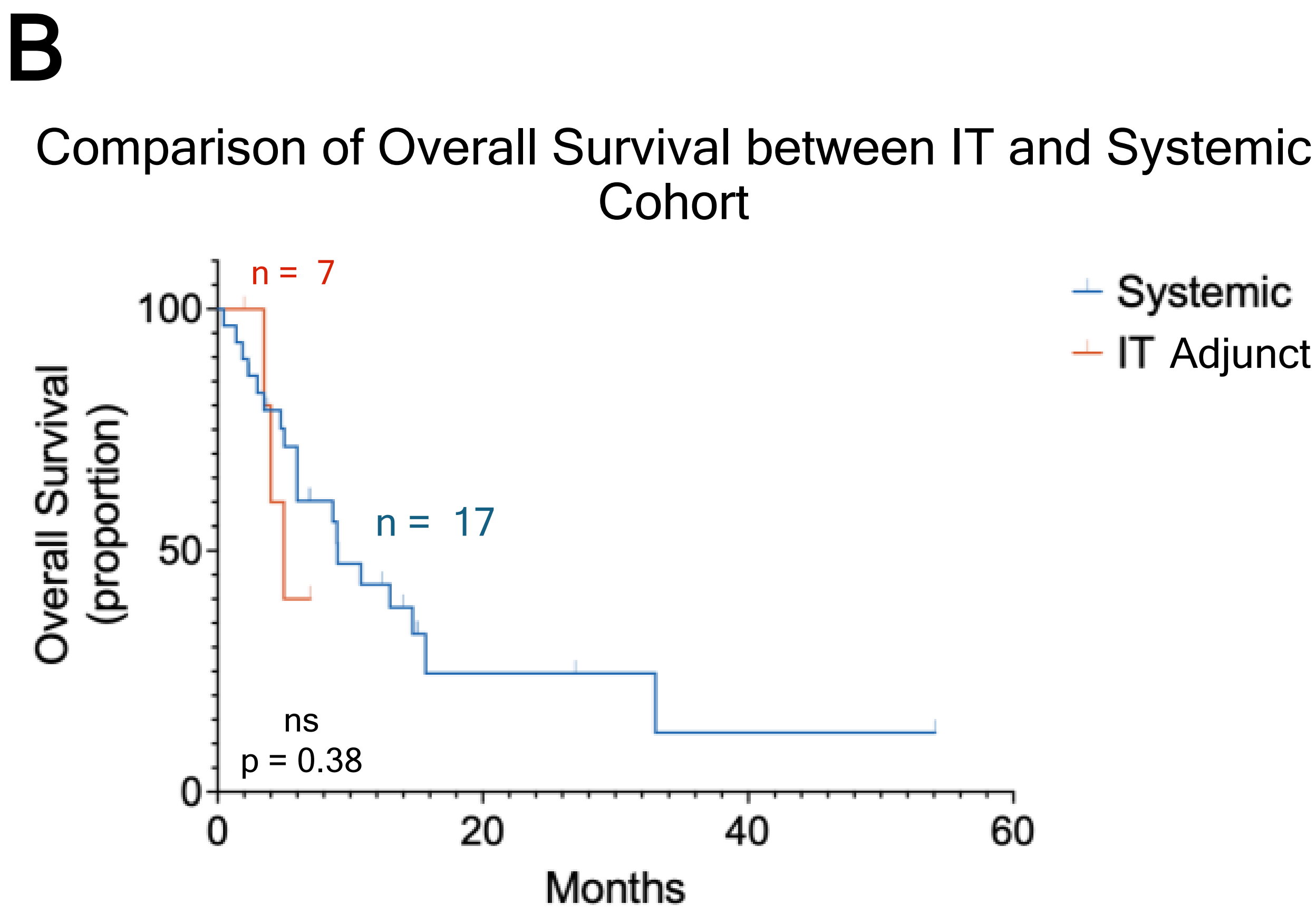

Number at Risk

Time (mos)	0	10	20	30	40	50	60
Systemic	17	5	1	1	0	0	0
IT Adjunct	7	0	0	0	0	0	0

Supplemental Figure 7 . Kaplan-Meier analysis of (A) Progression-Free Survival of Patient Receiving IT Adjunct versus Systemic ICI therapy (B) Overall Survival of Patient Receiving IT Adjunct versus Systemic ICI therapy

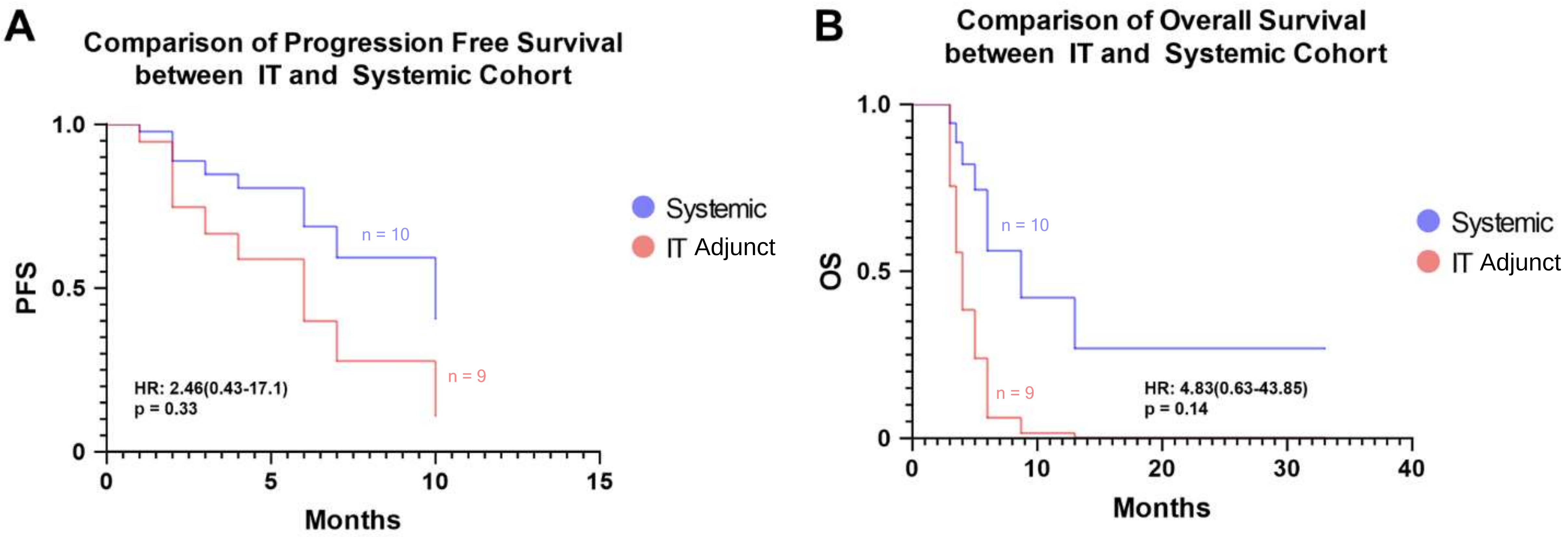

Supplemental Figure 8. Cox proportional hazards analysis of (A) progression-free survival (PFS) and (B) overall survival (OS) comparing patients receiving IT-Adjunct versus systemic immune checkpoint inhibitor (ICI) therapy.

*__Note:__ Multivariable-adjusted models were limited by covariate availability, resulting in a reduced sample size compared to unadjusted analyses
