# Supplementary material for: Intrathecal immune checkpoint inhibitors as adjunctive therapy for leptomeningeal disease: A case series and systematic review
Source: Neurosurg Rev. 2026 Aug 21;49(1):531. doi: 10.1007/s10143-026-04454-z (PMC13493459; doi:10.1007/s10143-026-04454-z)
Supplement: Supplementary file 3 [file 10143_2026_4454_MOESM3_ESM.pdf]

Author - Year	Study Design	Primary Tumors No. patients (%)	ICI agents No. Patients (%)	Concomitant LMD Treatments No. Patients (%)	Interval between Primary and LMD months, median (range)	Duration of ICI months, Median (Range)	Duration between LMD Dx and ICI Use months, Median (Range)	LMD Radiological Response No. Patients (%)	Adverse Events No. Patients (%)	> G3 Adverse Events No. Patients (%)	PFS from start ICI months, Median (Range)	LMD OS	ICI Dosing Information
Alhusaini 2024[1]	Case Report	1 Melanoma (100)	1 Ipi+Nivo (100)	0	NR	1.61	0	1 PD (100)	NR	NR	1.61	12.4	Ipi + Nivo IV q3w ×4 → Nivo IV q2w (maintenance)
Aw 2023[2]	Case Report	1 NSCLC (100)	1 Pem (100)	0	1	13	0	1 CR (100)	1 (100)	0 (0)	10	13	Pembrolizumab IV (NR dosing schedule)
Bonomi 2020[3]	Case Report	1 Clear Cell Kidney Cancer (100)	1 Nivo (100)	0	NR	27	2	1 CR (100)	0 (0)	0 (0)	27	27	Nivolumab 3 mg/kg IV q2w
Brastianos et al. [4]	Single Arm Phase II Clinical Trial	17 Breast Cancer (85)<br>1 NSCLC (5)<br>1 SCLC (5)<br>1 Ovarian (5)	20 Pem (100)	0	39.5 (3-202)	NR	1.9 (0.3-23.6)	11 SD (55)<br>5 PD (25)<br>4 NA (20)	19 (95)	8 (40)		3.6 (2.2-5.2)	Pembrolizumab 200 mg IV q3w
Brastianos et al. [5]	Single Arm Phase II Clinical Trial	8 Breast Cancer (44.4)<br>2 Melanoma (11.1)<br>1 Anaplastic Astrocytoma (5.6)<br>1 Esophageal Adenocarcinoma (5.6)<br>1 Ependymoma (5.6)<br>1 GE Junction Adenocarcinoma (5.6)<br>1 Glioblastoma (5.6)<br>1 Lung Adenocarcinoma (5.6)<br>1 Ovarian (5.6)<br>1 SCLC (5.6)	18 Ipi+Nivo (100)	0	29.5 (3-276)	NR	NR	1 CR (5.6)<br>7 SD (38.9)<br>4 PD (22.2)<br>6 NA (33.3)	16 (88.9)	6 (33)	1.93 (1.28-2.66)	2.9 (1.6-5.0)	Nivolumab 1 mg/kg IV + Ipilimumab 3 mg/kg IV q3w × 4 → followed by nivolumab maintenance
Chorti 2021[6]	Retrospective Review	52 Melanoma (100)	19 PD-1i (25)	3 B/M (15.8)<br>1 B/M+RT (5.3)<br>9 RT (47.4)	8.5 (0-91.5)	NR	NR	NR	NR	NR	NR		PD-1 inhibitor regimens varied (NR standardized dosing)

Chu 2023[7]	Case Series	1 Cholangiocarcinoma (16.7)<br>2 Lung Cancer (33.3)<br>1 Bladder Cancer (16.7)<br>1 Gastric Cancer (16.7)<br>1 Unknown (16.7)	4 Nivo (66.7)<br>2 Tori (33.3)	5 SCS (83.3)<br>1 WBRT (16.7)	NR	NR	NR	NR	NR	NR	4.89 (1.87-15.02	7.0 (1.87-15.1)	Nivolumab 3 mg/kg IV q2w; Toripalimab 240 mg IV q3w
Fedorenko 2016 [8]	Case Report	1 Uveal Melanoma (100)	1 Ipi (100)	0	24	2	2	NR	NR		4	6	Ipilimumab 3 mg/kg IV q3w
Ferguson 2019[9]	Retrospective Review	178 Melanoma (100)	12 NR (6.7)	NR	48	NR	NR	NR	NR				ICI regimens varied (NR standardized dosing)
GeukesFoppen 2016 [10]	Retrospective Review	39 Melanoma (100)	10 Ipi (25.6)	2 Bi (20)<br>4 WBRT (30)<br>2 B/M + WBRT (20)	38.4 (0-348)	NR	NR	NR	NR				Ipilimumab regimens varied (NR standardized dosing)
Gion 2017 [11]	Case Report	1 Lung Adenocarcinoma (100)	1 Nivo (100)	0	0	7	9	1 SD (100)	0		7	7	Nivolumab 3 mg/kg IV q2w
Glitza 2018 [12]	Case Series	1 Uveal Melanoma (100)	1 Ipi (100)	1 WBRT, TMX, IT IL2 (100)	180	NR	NR	NR	NR		NR	9	Ipilimumab 3 mg/kg IV q3w
Lai 2024 [13]	Case Report	1 Lung Adenocarcinoma (100)	1 Pem (100)	1 Bevacizumab + Premetrexed (100)	31	6	17	1 SD (100)	NR		6	33	Pembrolizumab 100 mg IV q3w
Liu 2023 [14]	Retrospective Review	97 NSCLC (100)	16 Unknown (16.5)	NR	NR	NR	NR	NR	NR		NR	NR	ICI regimens varied (NR standardized dosing)
Mills 2024 [15]	Retrospective Review	8 Lung Adenocarcinoma (88.9)<br>1 Squamous Cell Carcinoma of Lung (11.1)	3 Avel (33.3)<br>6 Nivo (66.7)<br>4 Pem (44.4)	NR	NR	NR	NR	NR	NR		NR	NR	Nivolumab, pembrolizumab, or avelumab (standard dosing; regimen not specified)
Naidoo et al [16]	Single Arm Phase II Clinical Trial	3 NSCLC (23.1)<br>1 HNSCC (7.6)<br>1 SCC of Skin (7.6)<br>5 Breast Carcinoma (7.6)<br>3 HGG (23.1)	13 Pem (100)	0	NR	NR	NR	2 CR (15)<br>3 SD (23)<br>8 PD (62)	5	2	2.9 (1.1-NR)	4.9 (3.7-NR)	Pembrolizumab 200 mg IV q3w

Pabon 2024 [17]	Case Series	2 Esophageal Adenocarcinoma (100)	1 Ipi + Nivo (50)<br>1 Pem (50)	2 IT Topotecan (100)<br>1 WBRT + SRT (50)	10 (NR-10)	10 (6-14)	1	2 CR (100)	0	0	10 (6-14)	10 (6-14)	Nivolumab + ipilimumab (240 mg IV q2W + 1 mg/kg q6W) pembrolizumab (200 mg IV q3W)
Schwartz 2018 [18]	Case Report	1 Glioblastoma (100)	1 Pem (100)	1 Beavcizumab (100)	8	2	5	1 SD (100)	0		2	8.7	Pembrolizumab 200 mg IV q3w
Sener 2024 [19]	Case Report	2 Melanoma (100)	1 Rela (50)<br>1 Ipi (50)<br>1 Nivo (50)<br>1 Ipi + Nivo (50)	1 B/M, WBRT, SCS Bevacizumab (50)<br>1 WBRT	65.2 (26.6-103.8)	4.65 (2.4-6.9)	0.95 (0-1.9)	NR	1 (50)		4.65 (2.4-6.9)	6.15 (5.4-6.9)	ICI regimens varied (NR standardized dosing);
Tam 2024 [20]	Case Report	1 Breast Cancer (100)	1 Atez (100)	1 Capectiabine + IT Topotecan (100)	NR	NR	NR	NR	NR		NR	NR	Atezolizumab IV, NR dose/schedule; given with capecitabine and weekly IT topotecan after WBRT
Tetu 2020 [21]	Retrospective Review	29 Melanoma (100)	24 Unknown (82.8)	5 Bi (20.8)	NR	Nr	NR	NR	NR		NR	5.1 (2.43-15.67)	ICI regimens varied: ipilimumab, anti-PD-1, nivolumab + ipilimumab, or anti-PD-1 + BRAF inhibitor; NR standardized dosing
Wang 2021 [22]	Case Report	1 Primary Hepatic NET (100)	1 Nivo (100)	1 WBRT (100)	24	3	0	1 CR (100)	1		3	3	Nivolumab 200 mg IV per dose ×2 cycles over 3 weeks; later 2 additional cycles

Supplementary Table 1. Characteristics of Included Studies of Systemic Immune Checkpoint Inhibitor Therapy for Leptomeningeal Disease

Author - Year	Study Design	Primary Tumors No. patients (%)	ICI agents No. Patients (%)	Concomitant LMD Treatments No. Patients (%)	Interval between Primary and LMD months, median (range)	Duration of ICI months, Median (Range)	Duration between LMD Dx and ICI Use months, Median (Range)	LMD Radiological Response No. Patients (%)	Adverse Events No. Patients (%)	> G3 Adverse Events No. Patients (%)	PFS from start ICI months, Median (Range)	LMD OS	ICI Dosing Infromation
Chevallier 2024 [23]	Case Report	1 Melanoma (100)	1 Nivo (100)	1 Systemic TT (100)	107	7	0	1 CR (100)	NR		7	7	IT nivolumab 50 mg q2w; concurrent encorafenib + binimetinib at full dose
Dan 2024 [24]	Case Report	1 Melanoma (100)	1 Pem (100)	1 IV Pem (100)	19	1.5	2	NR	0	0	1.5	3.5	IT pembrolizumab 25 mg + pembrolizumab 175 mg IV q3w ×2 cycles; concurrent dabrafenib + trametinib
Ensign 2021 [25]	Case Report	1 Breast Cancer (100)	1 Pem (100)	1 IV Pem (100)	48	2	2	NR	0	0	2	4	IT pembrolizumab 5 mg q3w ×2 cycles; concurrent systemic pembrolizumab 200 mg IV q3w
Huppert 2020 [26]	Case Report	2 Melanoma (100)	2 Nivo (100)	1 IV Nivo (50)<br>1 B/M (50)	78 (72-84)	1.5 (1-2)	1.5 (0-3)	1 PR (50)<br>1 PD (50)	1 (50)	0	1.5 (1-2)	3.5 (2-5)	Patient 1: Nivolumab 240 mg IV + IT nivolumab 20 mg q14d Patient 2: IT nivolumab 20 mg q2w; concurrent dabrafenib + trametinib

Glitza-Olivia 2023[27]	Single Arm Phase I Clinical Trial	25 Melanoma (100)	25 Nivo (100)	25 IV Nivo (100)<br>11 B/M (44)	NR	NR	1.63 (0-22.2)	NR	10	0	NR	NR	IT nivolumab 5, 10, 20, or 50 mg q2w; IV nivolumab 240 mg q2w from cycle 2 onward
Zhen 2024[28]	Retrospective Review	8 Melanoma (100)	8 anti-PD-1i (100)	3 RT (37.5)<br>6 IV anti-PD-1 (75)<br>4 B/M (50)<br>3 anti-CTLA-4 (37.5)<br>2 TMZ (25)<br>2 Bevacicumab (25)<br>2 Nab-paclitaxel (25)	NR	NR	NR	2 PR (25)<br>2 SD (25)	5	0	3.7 (2.28-NA)	4.86 (3.06-NA)	IT nivolumab 20 mg q2w or intrathecal pembrolizumab 20 mg q3w; concurrent systemic therapy varied
Institutional Patient 1	Institutional	1 Melanoma (100)	1 Nivo (100)	1 B/M (100)<br>1 IV Nivo (100)	24	4	0	NR	1 (100)	0	4	5	IT nivolumab 50 mg ×1 dose; later IV nivolumab + relatlimab ×2 cycles
Institutional Patient 2	Institutional	1 Melanoma (100)	1 Nivo (100)		79	2	0	NR	0 (0)	0	2	2	IT nivolumab 50 mg q3w ×3 doses; concurrent IV nivolumab 150 mg

Supplementary Table 2. Characteristics of Included Studies of Intrathecal Adjunct Immune Checkpoint Inhibitor Therapy for Leptomeningeal Disease

References

1. Alhusaini S, Naya L, Reddy SA, Patel CB. MEK Pathway Inhibitor-Mediated Response in BRAF V600-Mutant Melanoma with Brain Parenchymal and Leptomeningeal Metastases. *Annals of Neurology*. 2024;96(6):1227-1229. doi:10.1002/ana.27062
2. Aw YTV, Peters GD. Durable complete leptomeningeal and intracerebral responses to first-line Pembrolizumab in highly-selected Non-small cell lung cancer patients. *Current Problems in Cancer: Case Reports*. 2023;9doi:10.1016/j.cpccr.2022.100209
3. Bonomi L, Bettini AC, Arnoldi E, et al. Nivolumab efficacy in leptomeningeal metastasis of renal cell carcinoma: a case report. *Tumori*. Dec 2020;106(6):Np76-np78. doi:10.1177/0300891620904411
4. Brastianos PK, Lee EQ, Cohen JV, et al. Single-arm, open-label phase 2 trial of pembrolizumab in patients with leptomeningeal carcinomatosis. *Nat Med*. Aug 2020;26(8):1280-1284. doi:10.1038/s41591-020-0918-0
5. Brastianos PK, Strickland MR, Lee EQ, et al. Phase II study of ipilimumab and nivolumab in leptomeningeal carcinomatosis. *Nat Commun*. Oct 12 2021;12(1):5954. doi:10.1038/s41467-021-25859-y
6. Chorti E, Kebir S, Ahmed MS, et al. Leptomeningeal disease from melanoma—Poor prognosis despite new therapeutic modalities. *European Journal of Cancer*. 2021;148:395-404. doi:10.1016/j.ejca.2021.02.016
7. Chu Z, Lin H, Zhan Q, Liu T, Wang Y. A retrospective analysis of first-line PD-1 monoclonal antibodies treatment in patients with leptomeningeal metastasis from solid tumors. Article. *Journal of Oncology Pharmacy Practice*. 2023;29(3):582-589. doi:10.1177/10781552221074622
8. Fedorenko IV, Evernden B, Kenchappa RS, et al. A rare case of leptomeningeal carcinomatosis in a patient with uveal melanoma: Case report and review of literature. *Melanoma Research*. 2016;26(5):481-486. doi:10.1097/CMR.0000000000000274
9. Ferguson SD, Bindal S, Bassett RL, et al. Predictors of survival in metastatic melanoma patients with leptomeningeal disease (LMD). *Journal of Neuro-Oncology*. 2019;142(3):499-509. doi:10.1007/s11060-019-03121-2
10. Geukes Foppen MH, Brandsma D, Blank CU, van Thienen JV, Haanen JB, Boogerd W. Targeted treatment and immunotherapy in leptomeningeal metastases from melanoma. *Annals of Oncology*. 2016;27(6):1138-1142. doi:10.1093/annonc/mdw134
11. Gion M, Remon J, Caramella C, Soria JC, Besse B. Symptomatic leptomeningeal metastasis improvement with nivolumab in advanced non-small cell lung cancer patient. *Lung Cancer*. 2017;108:72-74. doi:10.1016/j.lungcan.2017.02.022
12. Glitza IC, Reddy ST, Patel SP. Leptomeningeal disease in uveal melanoma: a case series. Letter. *Journal of Neuro-Oncology*. 2018;139(2):503-505. doi:10.1007/s11060-018-2878-5
13. Lai M, Mu T, Liu M, et al. Personalized therapy guided by longitudinal liquid biopsies for treatment of leptomeningeal disease from lung adenocarcinoma: A case report. Article. *Oncology Letters*. 2024;28(1)doi:10.3892/ol.2024.14432
14. Liu X, Li G, Zhang H, et al. Molecular characteristics and prognostic factors of leptomeningeal metastasis in non-small cell lung cancer. *Clinical Neurology and Neurosurgery*. 2023;225doi:10.1016/j.clineuro.2022.107572
15. Mills MN, Uno A, Li P, et al. Clinical Outcomes of Patients with Non-Small Cell Lung Cancer Leptomeningeal Disease Following Receipt of EGFR-Targeted Therapy, Immune-

Checkpoint Blockade, Intrathecal Chemotherapy, or Radiation Therapy Alone. *Clinical Lung Cancer*. 2024;25(5):417-423.e1. doi:10.1016/j.cllc.2024.04.005
16. Naidoo J, Schreck KC, Fu W, et al. Pembrolizumab for patients with leptomeningeal metastasis from solid tumors: efficacy, safety, and cerebrospinal fluid biomarkers. *J Immunother Cancer*. Aug 2021;9(8)doi:10.1136/jitc-2021-002473
17. Pabon CM, Yeboa DN, O'Brien BJ, Majd NK, Wang C, Murphy MAB. Intrathecal topotecan with systemic checkpoint inhibitor therapy for gastroesophageal cancer with leptomeningeal involvement: two case reports and review of the literature. Article. *Journal of Gastrointestinal Oncology*. 2024;15(3):1331-1340. doi:10.21037/jgo-24-70
18. Schwartz C, Romagna A, Machegger L, et al. Extensive Leptomeningeal Intracranial and Spinal Metastases in a Patient with a Supratentorial Glioblastoma Multiforme, IDH-Wildtype. *World Neurosurgery*. 2018;120:442-447. doi:10.1016/j.wneu.2018.09.082
19. Sener U, Webb M, Breen WG, et al. Proton Craniospinal Irradiation with Immunotherapy in Two Patients with Leptomeningeal Disease from Melanoma. *Journal of Immunotherapy and Precision Oncology*. 2024;7(1):1-6. doi:10.36401/JIPO-23-20
20. Tam A, Li YR, Williams T, Yoon S. Grade 5 Radiation Necrosis After Whole-Brain Radiation Therapy. *Practical Radiation Oncology*. 2024;14(2):87-92. doi:10.1016/j.prro.2023.10.012
21. Tétu P, Sirven-Villaros L, Cuzzubbo S, et al. Impact of new systemic treatment and radiotherapy in melanoma patients with leptomeningeal metastases. *Cancers*. 2020;12(9):1-14. doi:10.3390/cancers12092635
22. Wang WJ, Lee KD, Lu LS, Ting LL, Chiou JF. Treating leptomeningeal metastases from primary hepatic neuroendocrine carcinoma with combined radiotherapy and immunotherapy: A case report. *Therapeutic Radiology and Oncology*. 2021;5(March)doi:10.21037/tro-19-103
23. Chevallier E, Flory V, Chassang M, et al. Concurrent intrathecal nivolumab and targeted therapy in a patient with melanoma-associated leptomeningeal disease. *J Eur Acad Dermatol Venereol*. Sep 12 2024;doi:10.1111/jdv.20336
24. Dan X, Huang M, Sun Z, Chu X, Shi X, Chen Y. Case report: Concurrent intrathecal and intravenous pembrolizumab for metastatic melanoma with leptomeningeal disease. *Frontiers in Oncology*. 2024;14doi:10.3389/fonc.2024.1344829
25. Ensign SPF, Yancey E, Anderson KS, Mrugala MM. Safety and feasibility of intrathecal pembrolizumab infusion in refractory triple negative breast cancer with leptomeningeal disease: A case report. *Current Problems in Cancer: Case Reports*. 2021;4doi:10.1016/j.cpccr.2021.100103
26. Huppert LA, Melisko ME, Glastonbury CM, Khanafshar E, Daud AI. Treatment of metastatic melanoma with leptomeningeal disease using intrathecal immunotherapy. *JCO Oncology Practice*. 2020;16(11):757-759. doi:10.1200/OP.20.00146
27. Glitza Oliva IC, Ferguson SD, Bassett R, et al. Concurrent intrathecal and intravenous nivolumab in leptomeningeal disease: phase 1 trial interim results. *Nature Medicine*. 2023;29(4):898-905. doi:10.1038/s41591-022-02170-x
28. Zhen J, Chen L, Wang H, et al. Intrathecal anti-PD-1 treatment in metastatic melanoma patients with leptomeningeal disease (LMD): real-world data and evidence. *Journal of Neuro-Oncology*. 2024;170(3):665-673. doi:10.1007/s11060-024-04843-8
